# Supplementary material for: The effect of farmland on the surface water of the Aral Sea Region using Multi-source Satellite Data
Source: PeerJ. 2022 Feb 10;10:e12920. doi: 10.7717/peerj.12920 (PMC8841034; doi:10.7717/peerj.12920)
Supplement: Supplemental Information 1 [file peerj-10-12920-s001.docx]

**Table S1.** Operating parameters of LandTrendr

| **Parameter** | **Value** |
| --- | --- |
| Spectral Index | NDVI |
| Max segments | 6 |
| Pval | 0.05 |
| Recovery threshold | 0.25 |
| Despike | 0.9 |
| Optimal model scale | 0.75 |
