# Supplementary material for: The effect of farmland on the surface water of the Aral Sea Region using Multi-source Satellite Data
Source: PeerJ. 2022 Feb 10;10:e12920. doi: 10.7717/peerj.12920 (PMC8841034; doi:10.7717/peerj.12920)
Supplement: Supplemental Information 2 [file peerj-10-12920-s002.docx]

**Table S2.** Variables of Random Forest Algorithm

| **Feature Collection** | **Variable** |
| --- | --- |
| Landsat 5 | Blue, Green, Red, Near infrared, Shortwave infrared 1, Thermal Infrared, Shortwave infrared 2, NDVI |
| Landsat 7 | Blue, Green, Red, Near infrared, Shortwave infrared 1, Brightness temperature, Shortwave infrared 2, NDVI |
| Landsat 8 | Coastal aerosol, Blue, Green, Red, Near infrared, Shortwave infrared 1, Shortwave infrared 2, NDVI |
