# Supplementary material for: The effect of farmland on the surface water of the Aral Sea Region using Multi-source Satellite Data
Source: PeerJ. 2022 Feb 10;10:e12920. doi: 10.7717/peerj.12920 (PMC8841034; doi:10.7717/peerj.12920)
Supplement: Supplemental Information 3 [file peerj-10-12920-s003.docx]

**Table S3.** Confusion matrix for the 1987 classified image.

| **Validation Data** | | | | | | |
| --- | --- | --- | --- | --- | --- | --- |
| **Class** | **Water** | **Farmland** | **Irrigation Land** | **Shoal** | **Desert** | **UA** |
| **Water** | 198 | 15 | 0 | 0 | 1 | 92.5% |
| **Farmland** | 2 | 172 | 10 | 4 | 8 | 87.8% |
| **Irrigation land** | 0 | 13 | 190 | 0 | 0 | 94.0% |
| **Shoal** | 0 | 0 | 0 | 181 | 11 | 94.3% |
| **Desert** | 0 | 0 | 0 | 15 | 180 | 92.3% |
| **Total** | 200 | 200 | 200 | 200 | 200 | OA:92.1% |
| **PA** | 99.0% | 86.0% | 95.0% | 90.5% | 90.0% |  |
