# Supplementary material for: The effect of farmland on the surface water of the Aral Sea Region using Multi-source Satellite Data
Source: PeerJ. 2022 Feb 10;10:e12920. doi: 10.7717/peerj.12920 (PMC8841034; doi:10.7717/peerj.12920)
Supplement: Supplemental Information 4 [file peerj-10-12920-s004.docx]

**Table S4.** Confusion matrix for the change detection of abandoned farmland.

| **Class** | **Stable Region** | **Changed Region** | **Total** | **PA** |
| --- | --- | --- | --- | --- |
| **Stable Region** | 188 | 47 | 235 | 80.0% |
| **Changed Region** | 12 | 153 | 165 | 92.7% |
| **Total** | 200 | 200 | OA:85.3% | |
| **UA** | 94.0% | 76.5% |  |  |
