# Supplementary material for: The effect of farmland on the surface water of the Aral Sea Region using Multi-source Satellite Data
Source: PeerJ. 2022 Feb 10;10:e12920. doi: 10.7717/peerj.12920 (PMC8841034; doi:10.7717/peerj.12920)
Supplement: Supplemental Information 5 [file peerj-10-12920-s005.docx]

**Table S5.** Area of Abandoned Farmland near the Amu Draya and the Syr Draya

| **Distance (km)** | **Amu Darya (km2)** | **Syr Darya (km2)** | **Total (km^2^)** |
| --- | --- | --- | --- |
| 1 | 60.566 | 18.902 | 79.468 |
| 3 | 154.931 | 73.973 | 228.904 |
| 5 | 273.618 | 120.765 | 394.383 |
| 7 | 373.885 | 153.169 | 527.054 |
| 9 | 470.358 | 176.082 | 646.440 |
