# Supplementary material for: The effect of farmland on the surface water of the Aral Sea Region using Multi-source Satellite Data
Source: PeerJ. 2022 Feb 10;10:e12920. doi: 10.7717/peerj.12920 (PMC8841034; doi:10.7717/peerj.12920)
Supplement: Supplemental Information 6 [file peerj-10-12920-s006.docx]

**Table S6.** Area of Abandoned Farmland in the Aral Sea Region

| **Year** | **Abandoned Farmland** | |
| --- | --- | --- |
|  | **Area (km^2^)** | **Percentage (%)** |
| 1988 | 84 | 2.7 |
| 1989 | 226 | 7.2 |
| 1990 | 173 | 5.5 |
| 1991 | 232 | 7.4 |
| 1992 | 257 | 8.2 |
| 1993 | 139 | 4.5 |
| 1994 | 65 | 2.1 |
| 1995 | 113 | 3.6 |
| 1996 | 111 | 3.5 |
| 1997 | 51 | 1.6 |
| 1998 | 71 | 2.3 |
| 1999 | 104 | 3.3 |
| 2000 | 161 | 5.1 |
| 2001 | 72 | 2.3 |
| 2002 | 76 | 2.4 |
| 2003 | 47 | 1.5 |
| 2004 | 60 | 1.9 |
| 2005 | 53 | 1.7 |
| 2006 | 103 | 2.3 |
| 2007 | 69 | 2.2 |
| 2008 | 63 | 2 |
| 2009 | 45 | 1.5 |
| 2010 | 72 | 2.3 |
| 2011 | 76 | 2.4 |
| 2012 | 35 | 1.1 |
| 2013 | 81 | 2.6 |
| 2014 | 38 | 1.2 |
| 2015 | 45 | 1.5 |
| 2016 | 55 | 1.8 |
| 2017 | 177 | 5.7 |
| 2018 | 106 | 3.5 |
| 2019 | 100 | 3.2 |
| **Total** | **3129** | **100** |
