# Supplementary material for: The effect of farmland on the surface water of the Aral Sea Region using Multi-source Satellite Data
Source: PeerJ. 2022 Feb 10;10:e12920. doi: 10.7717/peerj.12920 (PMC8841034; doi:10.7717/peerj.12920)
Supplement: Supplemental Information 7 [file peerj-10-12920-s007.docx]

**Table S7.** Comprehensive evaluation index system of water and farmland coupling degree.

| **Evaluation System** | **Subsystem** | **Index** | **Symbol** | **Unit** |
| --- | --- | --- | --- | --- |
| Water-Farmland | Water System | Water Volume of Aral Sea | A1 | km^3^ |
|  |  | Water Storage of the Aral Sea Region | A2 | km^3^ |
|  | Farmland System | Area of Abandoned Farmland | B1 | km^2^ |
