# Supplementary material for: The effect of farmland on the surface water of the Aral Sea Region using Multi-source Satellite Data
Source: PeerJ. 2022 Feb 10;10:e12920. doi: 10.7717/peerj.12920 (PMC8841034; doi:10.7717/peerj.12920)
Supplement: Supplemental Information 8 [file peerj-10-12920-s008.docx]

**Table S8.** Weight of every index, coupling degree value of every year and average coupling degree value.

| **Subsystem** | **Index** | **Weight** | **Year** | **Coupling Degree Value** | **Average Coupling Degree Value** |
| --- | --- | --- | --- | --- | --- |
| Water System | A1 | 0.141 | 2003 | 0.787 | 0.903 |
|  |  |  | 2004 | 0.895 |  |
|  | A2 | 0.759 | 2005 | 0.774 |  |
| Farmland System | B1 | 0.1 | 2006 | 0.979 |  |
|  |  |  | 2007 | 0.993 |  |
|  |  |  | 2008 | 0.923 |  |
|  |  |  | 2009 | 0.971 |  |
