# Supplementary material for: The effect of farmland on the surface water of the Aral Sea Region using Multi-source Satellite Data
Source: PeerJ. 2022 Feb 10;10:e12920. doi: 10.7717/peerj.12920 (PMC8841034; doi:10.7717/peerj.12920)
Supplement: Supplemental Information 9 [file peerj-10-12920-s009.docx]

**Table S9.** Criteria for Coupling Coordination Type

| **Coordination Type** | **Degree of Coordination** |
| --- | --- |
| high-quality coupling coordination | [0.9, 1) |
| good coupling coordination | [0.8, 0.9) |
| intermediate coupling coordination | [0.7, 0.8) |
| primary coupling coordination | [0.6, 0.7) |
| reluctant coupling coordination | [0.5, 0.6) |
| border imbalance | [0.4, 0.5) |
| mild imbalance | [0.3, 0.4) |
| intermediate imbalance | [0.2, 0.3) |
| serious imbalance | [0.1, 0.2) |
| extreme imbalance | (0, 0.1) |
