# Supplementary material for: The effect of farmland on the surface water of the Aral Sea Region using Multi-source Satellite Data
Source: PeerJ. 2022 Feb 10;10:e12920. doi: 10.7717/peerj.12920 (PMC8841034; doi:10.7717/peerj.12920)
Supplement: Supplemental Information 10 [file peerj-10-12920-s010.docx]

**Table S10.** Crop Water use efficiency in Aral Sea Region.

| **Time** | **Crop** | | **Crop Water Use Efficiency (**kg/km^3^**)** | **Data Source** |
| --- | --- | --- | --- | --- |
| 2000-2014 | Cotton | 4.51*10^8^ | | Food and Agriculture Organization of the United Nations |
|  | Wheat | 8.81*10^8^ | |  |
|  | Rice | 6.89*10^8^ | |  |
