# Supplementary material for: The effect of farmland on the surface water of the Aral Sea Region using Multi-source Satellite Data
Source: PeerJ. 2022 Feb 10;10:e12920. doi: 10.7717/peerj.12920 (PMC8841034; doi:10.7717/peerj.12920)
Supplement: Supplemental Information 11 [file peerj-10-12920-s011.docx]

**Table S11.** Crop production in the Aral Sea Region.

| **Year** | **Production of cotton (kg/km^2^)** | | **Production of wheat (kg/km^2^)** | **Production of rice (kg/km^2^)** | **Data Source** |
| --- | --- | --- | --- | --- | --- |
| 1992 | 247720 | 149745 | | 320290 | Food and Agriculture Organization of the United Nations, Statistical Yearbook, World Bank |
| 1993 | 249830 | 113815 | | 345310 |  |
| 1994 | 222795 | 113200 | | 297795 |  |
| 1995 | 235605 | 135845 | | 208395 |  |
| 1996 | 201260 | 137350 | | 227020 |  |
| 1997 | 216735 | 152215 | | 260560 |  |
| 1998 | 175150 | 157670 | | 286415 |  |
| 1999 | 206875 | 196640 | | 275215 |  |
| 2000 | 198485 | 181010 | | 324735 |  |
| 2001 | 225805 | 216560 | | 259850 |  |
| 2002 | 217315 | 256525 | | 295540 |  |
| 2003 | 203785 | 237825 | | 308440 |  |
| 2004 | 227695 | 229445 | | 313175 |  |
| 2005 | 246220 | 257765 | | 331175 |  |
| 2006 | 235145 | 267280 | | 349465 |  |
| 2007 | 238735 | 288845 | | 374430 |  |
| 2008 | 221310 | 272430 | | 336875 |  |
| 2009 | 225115 | 304770 | | 399895 |  |
| 2010 | 217720 | 266680 | | 378975 |  |
| 2011 | 240555 | 310845 | | 444975 |  |
| 2012 | 263430 | 275120 | | 402005 |  |
| 2013 | 272160 | 290775 | | 571365 |  |
| 2014 | 256370 | 293610 | | 562720 |  |
| 2015 | 267195 | 300285 | | 515725 |  |
| 2016 | 247740 | 300670 | | 552180 |  |
| 2017 | 240770 | 277960 | | 511540 |  |
| 2018 | 232695 | 267705 | | 500270 |  |
| 2019 | 259460 | 281950 | | 517250 |  |
