# Supplementary material for: The effect of farmland on the surface water of the Aral Sea Region using Multi-source Satellite Data
Source: PeerJ. 2022 Feb 10;10:e12920. doi: 10.7717/peerj.12920 (PMC8841034; doi:10.7717/peerj.12920)
Supplement: Supplemental Information 12 [file peerj-10-12920-s012.docx]

**Table S12.** Water Consumption for Planting Cotton in Abandoned Farmland.

| **Year** | **Water Volume (km^3^)** |
| --- | --- |
| 2000 | 0.070 |
| 2001 | 0.036 |
| 2002 | 0.012 |
| 2003 | 0.021 |
| 2004 | 0.030 |
| 2005 | 0.028 |
| 2006 | 0.053 |
| 2007 | 0.036 |
| 2008 | 0.03 |
| 2009 | 0.022 |
| 2010 | 0.010 |
| 2011 | 0.013 |
| 2012 | 0.020 |
| 2013 | 0.018 |
| 2014 | 0.021 |
| Average | 0.028 |
